# Gene action, combining ability, heterotic grouping and hybrid performance of tropical maize inbred lines from a public breeding program in Brazil

**DOI:** 10.3389/fpls.2026.1890118

**Published:** 2026-09-08

**Authors:** Gabriela dos Santos Pereira, Victória Manhago Salvador, Yuri Mike Pontes Belo Lança, Rhai Christy Alves Silva, Mateus Feliciano Bicalho, Letícia Prada de Miranda, Rodrigo Oliveira DeLima

**Affiliations:** 1 Department of Agronomy, Federal University of Viçosa, Viçosa, Brazil; 2 Limagrain, Petrolina, Pernambuco, Brazil; 3 Tropical Melhoramento e Genética, Sorriso, Mato Grosso, Brazil; 4 GDM Seeds, Londrina, Paraná, Brazil

**Keywords:** breeding strategies, diallel, genetic distance, heterotic patterns, inheritance, tropical environments, zea mays

## Abstract

**Introduction:**

Single-cross hybrids are the predominant type of cultivar planted by maize growers worldwide. The clear understanding of both the inheritance of target traits and the heterotic patterns of breeding germplasm is essential for the development of heterotic and high-yielding single-cross hybrids in maize breeding programs. Therefore, our objectives were to determine the inheritance of agronomic traits in improved tropical maize germplasm; estimate the general and specific combine abilities (GCA and SCA) effects for these traits among 15 selected inbred lines; group these lines into heterotic groups using two approaches; and identify high-yielding hybrids derived from these lines.

**Methods:**

We evaluated 105 F_1_ single-cross hybrids derived from crossing among the 15 selected inbred lines using diallel mating design, along with five checks commercial hybrids, for a wide range of agronomic traits over three summer seasons in southeastern Brazil. A mixed model approach was used to estimate the variance components of GCA and SCA, to predict the GCA and SCA effects, and also to obtain adjusted means of the hybrids.

**Results and discussion:**

We found that the inheritance of most traits is predominantly governed by additive gene action, whereas grain yield is largely modulated by non-additive gene action in our set of selected inbred lines of tropical maize. The lines VML016, VML062, VML081, VML083 and VML131 are promising for simultaneously improving GY and reducing cycle and plant stature, whereas the VML105 and VML157 should be used to reduce cycle and increase grain yield, respectively, in our maize breeding program. Both the genetic distance among lines and the SCA effects for GY are effective for grouping the improved breeding lines into genetically distinct groups. Also, molecular makers should be employed to support the allocation of inbred lines into heterotic groups and aid in the selection of the best parental combinations for the development of breeding populations and hybrids in improved tropical maize germplasm. Finally, we identified 13 outstanding experimental hybrids that must be evaluated to confirm the consistence and stability of their yield performance across a wider range of environments and management conditions in southeastern Brazil for future release and commercialization.

## 1 Introduction

Maize (*Zea mays* L.) is essential for human nutrition, livestock feed and biofuel production and is the largest cereal crop produced worldwide (Moro and Fritsche-Neto, 2017; Erenstein et al., 2022). Brazil is the world’s third-largest maize producer after the United States and China (Vieira et al., 2025; FAO, 2025; USDA, 2025). During the 2024/2025 growing season, Brazilian maize production reached almost 140 million tons of grain, harvested across an area of 22.5 million hectares (CONAB, 2025). Currently, hybrid maize accounts for over 95% of the maize production area in Brazil; and the single-cross hybrid is the most planted hybrid by Brazilian farmers (Kist et al., 2019). Consequently, all Brazilian breeding programs of maize have focused their efforts on the development of hybrids cultivars, mainly single-cross hybrids. The success of a maize hybrid breeding program depends on the key’s steps, including improvement of germplasm, development of breeding populations and inbred lines, selection of best lines, hybrid development of selected lines from opposite heterotic groups, identification of stable and high-yielding hybrids and commercial production of the best hybrids (Von Pinho et al., 2022; Andorf et al., 2019; Edmeades et al., 2017; Hallauer et al., 2010). Therefore, a deep understanding of the inheritance of grain yield (GY) and agronomic traits that influence yield performance, as well as the heterotic grouping of the breeding germplasm, is crucial for the development of high-yielding and stable maize hybrids.

The inheritance of GY and associated traits in tropical maize has been extensively studied over the past decade (Olajide et al., 2026; Akinyosoye et al., 2025; Bonkoungou et al., 2024; Beugnot et al., 2023; Owusu et al., 2023; Abu et al., 2021; Ribeiro et al., 2020; Ertiro et al., 2017; Badu-Apraku et al., 2016); and that the type of gene action governing GY and associated traits appears to depend on environmental conditions and the genetic background of the germplasm. In a pioneering study, Sprague and Tatum (1942) found that additive gene action is relatively more important than non-additive gene action in determining GY in unselected inbred lines, whereas non-additive gene action predominates in selected inbred lines of temperate maize. Similarly, Badu-Apraku et al. (2016) reported that non-additive gene action predominantly modulated the inheritance of GY, cycle, plant height and aspect, and kernel quality in a set of selected inbred lines of tropical maize, irrespective of environmental conditions. In another study, Oyekale et al. (2020) examined the inheritance of GY and related traits in tropical maize inbred lines derived from a broad-based population under stress (low-nitrogen and *Striga*-infested) and non-stress environments; and observed that additive gene action governed the traits inheritance under low-nitrogen, whereas both additive and non-additive gene action were important under non-stress and *Striga-*infested conditions. Recently, Adewale et al. (2023) found that GY inheritance in tropical maize lines also derived from a broad-based population was influenced by additive gene action under *Striga* infection and non-stress conditions, and by non-additive gene action under drought stress. However, the majority of studies have investigated the inheritance of agronomics traits in tropical maize lines derived from non-elite breeding populations with broader genetic base than those typically used in commercial hybrid breeding programs. Therefore, the inheritance of key agronomic traits in breeding inbred lines derived from elite and narrow-based populations in the commercial breeding programs is still poorly understood, hindering the adoption of suitable strategies to increase the rate of genetic gain. Additionally, most studies have focused on investigating the inheritance of GY, plant height and maturity; consequently, there is limited information on the inheritance of other yield-influencing traits, such as ear traits, kernel number, and both kernel size and weight in tropical maize, particularly within tropical maize breeding germplasm.

In any hybrid breeding program, the knowledge on heterotic grouping of the breeding germplasm is fundamental for guiding crosses in the development of breeding populations and highly heterotic and yielding hybrids, maximizing the usefulness of the parental lines (Arora et al., 2024; Hallauer et al., 2010; Fan et al., 2009, Fan et al., 2018; Reif et al., 2005). However, tropical maize inbred lines have been often derived from populations of mixed origin, including breeding populations obtained by selfing the commercial hybrids (Semagn et al., 2012; Trevisan, 2018; Wu et al., 2016). Commercial hybrids have been extensively used as germplasm source for developing new inbred lines in most maize breeding programs worldwide, especially in Brazil (Almeida et al., 2024; Luz et al., 2024; Leng et al., 2019; Faria et al., 2022; Guimarães et al., 2018). Consequently, most tropical maize lines consist of a mixture of heterotic groups and have a complex genetic background, challenging their effective utilization in hybrid development. Therefore, prior to their use in hybrid development, these inbred lines must be assigned to putative heterotic groups that can subsequently be improved by breeders through reciprocal pedigree recurrent selection schemes for combining ability (Faria et al., 2022; Li et al., 2022; Kolawole et al., 2019; Suwarno et al., 2014; Viana et al., 2013; Hallauer et al., 2010). Heterotic grouping of tropical maize germplasm has frequently been done using specific combining ability (SCA) effects for GY and/or molecular marker-based genetic distance (GD) among parental lines; however, studies have reported inconsistent results regarding the concordance and breeding efficiency of these approaches (Akinwale et al., 2014; Bonkoungou et al., 2024; Adewale et al., 2023; Badu-Apraku et al., 2013, Badu-Apraku et al., 2016). In a recent study, Annor et al. (2019) found that SCA for GY was the most reliable method for heterotic grouping of parental lines for the development of stable and high-yielding hybrids of tropical maize. Similarly, Olayiwola et al. (2021) observed that SCA for GY had the highest breeding efficiency for heterotic grouping of tropical inbred lines. In contrast, Adewale et al. (2023) found that heterotic grouping of tropical maize lines based on GD among them was the most efficient methods, suggesting that this approach should be adopted in tropical maize breeding programs to enhance breeding efficiency of hybrid development. Thus, further investigation into the assessment of heterotic orientation in tropical maize is necessary, particularly for breeding inbred lines derived from commercial hybrids, as limited information is available on the heterotic grouping of maize lines derived from commercial hybrids.

The maize breeding program from Universidade Federal de Viçosa (UFV), located in Viçosa (lat. 20°45’14’S; long. 42°52’55’’W; alt. 648 m a.s.l.), Minas Gerais state, was stablished in the early 1930s and therefore has nearly 100 years of history. Currently, it is the second largest and one of the most important public maize breeding programs in Brazil (Faria et al., 2022). Our main proposal is to develop and improve maize germplasm to be cultivated across tropical environments in Brazil, especially under drought and low-N and P stresses (Resende et al., 2026; Schuster et al., 2024; Uberti et al., 2023; Torres et al., 2019; Torres et al., 2018); and also train the next generation of maize breeders. Over two last decades, we developed a panel of almost 200 tropical maize inbred lines that represent the current UFV breeding pool (Faria et al., 2022). Recently, this inbred line panel was evaluated for agronomic traits based on testcross performance with three narrow-based testers across tropical environments in southeastern Brazil. Subsequently, the 15 best inbred lines were selected based on their testcross performance for grain yield and desirable maturity and plant stature. These selected inbred lines were crossed using a diallel mating design, and their hybrids were evaluated across tropical environments in southeastern Brazil. Therefore, our objectives were to: i) determine the inheritance patterns of a wide range of agronomic traits in improved tropical maize germplasm from a public breeding program in Brazil; ii) estimate the GCA and SCA effects for these traits among selected tropical maize inbred lines; iii) group the inbred lines into heterotic groups using both SNP-based GD among inbred lines and SCA effects for GY; and iv) evaluate the performance of inbred lines in hybrid combinations and identify high-yielding hybrids to be cropped across tropical environments in southeastern Brazil.

## 2 Materials and methods

### 2.1 Genetic materials

Fifteen tropical maize inbred lines were selected based on their *per se* performance for maturity, plant architecture traits, disease and pest tolerance, as well as their yield performance in testcrosses with three elite testers: the inbred lines VML024, VML090 and VML144 (Ribeiro, 2021; **Table 1**). The 15 selected inbred lines were crossed using a diallel mating design according to Griffing’s method 4 (Griffing, 1956) during the 2019 winter season. Each inbred line was used as both male and female, and seeds from reciprocal crosses were bulked to generate 105 F_1_ crosses (single-cross hybrids). Therefore, the estimation of reciprocal effects was not an objective of this study. All maize hybrids and inbred lines belong to the maize breeding program of the Department of Agronomy of Universidade Federal de Viçosa (UFV), Minas Gerais, Brazil.

**Table 1:** Description of the 15 tropical maize inbred lines used in our study.

Inbred line	Population source	Subpopulation^1^	Tester
VML081	A3663	2	VML024
VML083	30F53	3	
VML134	BRS1010	3	
VML154	DKB390	3	
VML176	5011	2	
VML055	P3041	2	VML090
VML062	Z8480	1	
VML105	C333	3	
VML140	Z8420	1	
VML157	A3663	3	
VML004	Balu184	2	VML144
VML016	DKB435	1	
VML066	P30F90	1	
VML115	AL30	1	
VML131	Z8447	1	

^1^ Subpopulations based on STRUCTURE software using SNPs markers (Faria et al., 2022).

### 2.2 Trial management and experimental design

The 105 single-cross hybrids obtained from diallel cross of the 15 inbred lines along with five checks commercial hybrids (total of 110 maize hybrids) were evaluated in the Experimental Station of UFV in Viçosa (20°45’S, 42°49’W, 661 m asl) during the 2021/22 summer season and in the Experimental Station of UFV in Coimbra (20°50’S, 42°48’W, 713 m asl) during the 2019/20, 2020/21 and 2021/22 summer seasons (total of four environments in southeastern Brazil). Although additional experiments were conducted in Viçosa, only one growing season was included in the analyses because the previous trials were excluded due to operational issues that compromised their experimental quality. All experiments were sown under no-tillage conditions. Fertilizer rates were the same and, in all environments, 100 kg ha^-1^ of P_2_O_5_, 60 kg ha^-1^ of K_2_O, and 20 kg ha^-1^ of N were applied before sowing. Urea (43% N) was applied as a top-dressing fertilizer at a rate of 380 kg ha^-1^ around the V4 and V6 growth stages (Abendroth et al., 2011). The experiments were conducted in rain-fed conditions, and no irrigation was applied. In all experiments, the weeds were controlled with pre-emergence herbicide followed by post-emergence herbicide applications using standard agronomic practices. Other trials management were the same for all experiments. Also, all maize seeds were treated similarly with CropStar^®^ (imidacloprid, thiocarb) and Maxim Advanced^®^ (metalaxyl-M, thiabendazole, fludioxonil).

In all environments, the 110 maize hybrids were laid out in an 11 x 10 alpha lattice incomplete block design with three replications. Each plot was 4.0 m row, with two rows spaced 0.80 m apart. Seeds were sown in late October 2019, 2020 and 2021. Initially, plots were over-seed with hand planters and then thinned at the V4 stage to stands of 62,500 plants ha^-1^.

### 2.3 Trait measurements

We measured a large set of traits related to plant architecture, yield components and grain morphology, and finally grain yield. For plant architecture, we measured eight traits: days to pollen (DTP), number of days from planting to 50% anthesis, defined as the date when 50% of the plants within a plot were shedding pollen; days to silking (DTS), number of days from planting to 50% silking, defined as the date when 50% of the plants within a plot exhibited visible silks; ear leaf area (LA, cm^2^), calculated using the following equation: LA = LW×LL×0.75 (Montgomery, 1909), where LA is leaf area, LW is leaf width, and LL is leaf length; plant height (PH, cm), distance from the soil surface to the ligule of the flag leaf; ear height (EH, cm), distance from the soil surface to the node of insertion of the uppermost ear; stalk diameter (SD, mm), measured at the first internode of the plant using a digital caliper; and above and below ear node number (AENN and BENN, respectively). For the yield components, we measured five traits: ear length (EL, cm), defined as the distance from the base of the ear to the last viable kernel; number of kernel rows (NKR); number of kernels per ear (NKE), calculated as the product of the number of kernels per row and the number of kernel rows per ear; ear diameter (ED, mm), measured at the midpoint of the ear using a digital caliper; and one thousand-kernel weight (TKW, g) as the weight of 1,000 kernels adjusted to 145 g kg^-1^ moisture. For the grain morphology, we measured five traits: grain perimeter (GP, mm), grain area (GA, mm^2^), grain length (GL, mm) and grain width (GW, mm). These last set of traits was measured by ImageJ (Schneider et al., 2012) using a sample of one hundred kernels of each plot. Finally, grain yield (GY) was recorded for all ears in the plots at physiological maturity. The ears were shelled, the grain weight and grain moisture percentage were recorded, and GY (kg ha^-1^) was calculated at 145 g kg^-1^ moisture.

### 2.4 Molecular markers

For molecular characterization, we genotyped the 15 inbred lines used in the diallel cross along with other inbred lines belonging to UFV maize breeding (Faria et al., 2022). Briefly, we obtained leaf tissue samples from the bulk of five plants for each inbred line and we sent them to DuPont Pioneer^®^ Company, where DNA extraction and genotyping were performed. All inbred lines were genotyped using the GoldenGate platform (Illumina, San Diego, CA, USA), containing 3,713 single SNPs distributed across 10 chromosomes (Fan et al., 2003). Out of the 3,713 SNPs genotyped, a set of 3,083 SNPs with a minor allele frequency (MAF) greater than 0.01 and a 90% call rate was selected for genetic characterization analysis of tropical maize inbred lines from UFV maize breeding (Faria et al., 2022).

### 2.5 Statistical analysis

#### 2.5.1 Analysis of hybrid performance

We performed a mixed model analysis for each agronomic trait across the four environments using the R package “ASReml” (Butler et al., 2018). Hybrid, environment and replication were considered fixed effects, whereas blocks within replications were considered random effects. The phenotypic values were modeled according to following Equation: ${\text{y}}_{\text{ijkl}}=\text{μ}+{\text{g}}_{\text{i}}+{\text{e}}_{\text{l}}+{\text{ge}}_{\text{il}}+{\text{r}}_{\left(\text{j}\right)\text{l}}+{\text{b}}_{\left(\text{k}\right)\text{jl}}+{\text{ϵ}}_{\text{ijkl}}$, where y_ijkl_ is the phenotypic value of i^th^ hybrid in k^th^ block within j^th^ replication at l^th^ environment; µ is the general mean; g_i_ is the fixed effect of i^th^ hybrid; e_l_ is the fixed effect of l^th^ environment; ge_il_is the fixed effect of genotype-by-environment interaction; r_(j)l_ is the fixed effect of j^th^ replication within l^th^ environment; b_(k)jl_ is the random effect of k^th^ block within j^th^ replication within l^th^ environment, with ${\text{b}}_{\left(\text{k}\right)\text{jl}}\sim \text{N}\left({0,\sigma }_{\text{b}}^{2}\right)$; and ε_iljk_ is the random effect of error, with $\epsilon_{\text{iljk}}\sim \text{N}\left({0,\sigma }_{\epsilon }^{2}\right)$. The Wald test was used to test fixed effects via the chi-square statistic (Gouriéroux et al., 1982), and a likelihood ratio test was used to test random effect of block via the chi-square statistics (Neyman and Pearson, 1928). Pearson’s correlation coefficients between tested traits were estimated based on adjusted means of experimental hybrids of each trait using the R package “stats” (R Core Team, 2022).

#### 2.5.2 Diallel analysis

We fitted a model for genomic best linear unbiased prediction (GBLUP) based on SNP average effects of substitution (α) and dominance deviations (δ) according to Equation: ${\text{y}}_{\text{ij}}=\mu +{\text{Z}}_{\alpha \text{ij}}\alpha +{\text{Z}}_{\delta \text{ij}}\delta +\epsilon_{\text{ij}}$, where y_ij_ is the genotypic value of SNP genotype A_i_A_j_ (i,j = 1,2); µ is the general mean; Z_α11_ = 2 – 2p for A_1_A_1_, Z_α12_ = 1 – 2p for A_1_A_2_, and Z_α22_ = -2p for A_2_A_2_; Z_δ11_ = -2q^2^ for A_1_A_1_, Z_δ12_ = 2pq for A_1_A_2_, and Z_δ22_ = -2p^2^ for A_2_A_2_, where p = frequency of A_1_ allele and q = frequency of A_2_ allele; and ϵ_ij_ is the effect of error of SNP genotype A_i_A_j_ (i,j = 1,2). We used a GBLUP parametrization, and the additive and dominance relationship matrices among 15 elite inbred lines of tropical maize were estimated from SNP markers based on method proposed by VanRaden (2008) and Vitezica et al. (2013), respectively.

In the first stage, we fitted a model for each trait in each environment and season according to the following Equation: ${\text{y}}_{\text{ijk}}=\text{u}+{\text{g}}_{\text{i}}+{\text{r}}_{\text{j}}+{\text{b}}_{\text{k}}+\epsilon_{\text{ijk}}$, where y_ijk_ is the phenotypic value of i^th^ hybrid within k^th^ block at j^th^ replication; u is the constant; g_i_ is the fixed effect of i^th^ hybrid; r_j_ is the fixed effect of j^th^ replication; b_k_ is the random effect of k^th^ block with ${\text{b}}_{\text{k}}\sim \text{N}\left({0,\sigma }_{\text{b}}^{2}\right)$, and ϵ_ijk_ is the random effect of error with $\epsilon_{\text{ijk}}\sim \text{N}\left({0,\sigma }_{\epsilon }^{2}\right)$. In the second stage, we fitted a joint diallel model for each trait across all environments using the adjusted mean of each hybrid estimated in the first stage and associated weights, according to the following Equation: ${\text{y}}_{\text{ijl}}=\mu +{\text{g}}_{\text{ij}}+{\text{s}}_{\text{ij}}+{\text{e}}_{\text{l}}+{\text{ge}}_{\text{ijl}}+{\text{se}}_{\text{ijl}}+\epsilon_{\text{ijl}}$, where y_ijl_ is the phenotypic value of hybrid combination between the i^th^ and j^th^ parents within l^th^ environment; μ is the general mean; g_ij_ is the random effect of GCA of i^th^ or j^th^ parent, with ${\text{g}}_{\text{ij}}\sim \text{N}\left(0,\sigma_{\text{GCA}}^{2}\right)$; s_ij_ is the random effect of SCA of hybrid combination between the i^th^ and j^th^ parents, with ${\text{s}}_{\text{ij}}\sim \text{N}\left(0,\sigma_{\text{SCA}}^{2}\right)$; e_l_ is the fixed effect of l^th^ environment; ge_ijl_ is the random effect of GCA x environment interaction, with ${\text{ge}}_{\text{ijl}}\sim \text{N}\left(0,\sigma_{\text{GCAxE}}^{2}\right)$; se_ijl_ is the random effect of SCA x environment interaction, with ${\text{se}}_{\text{ijl}}\sim \text{N}\left(0,\sigma_{\text{SCAxE}}^{2}\right)$; and ϵ_ijl_ is the random effect of error with $\epsilon_{\text{ijl}}\sim \text{N}\left(0,\sigma^{2}\right)$. The joint diallel analyses were performed based on the mixed models approach using the R package “ASReml” (Butler et al., 2018). A likelihood ratio test was used to test random effects via the chi-square statistics (Neyman and Pearson, 1928), and the Wald test was used to test fixed effects via the chi-square statistic (Gouriéroux et al., 1982).

Based on the variance components of GCA ( $\sigma_{\text{GCA}}^{2}$) and SCA ( $\sigma_{\text{SCA}}^{2}$) across environments, we estimated the additive ( $\sigma_{\text{A}}^{2}$) and dominance ( $\sigma_{\text{D}}^{2}$) components of genetic variance across environments as follows (Hallauer et al., 2010): $\sigma_{\text{A}}^{2}=2\sigma_{\text{GCA}}^{2}$ and $\sigma_{\text{D}}^{2}=\sigma_{\text{SCA}}^{2}$. Then, broad-sense heritability (H^2^) and narrow-sense (h^2^) were estimated as follows (Hallauer et al., 2010): ${\text{H}}^{2}=\frac{\left({\text{σ}}_{\text{A}}^{2}+{\text{σ}}_{\text{D}}^{2}\right)}{\left({\text{σ}}_{\text{A}}^{2}+{\text{σ}}_{\text{D}}^{2}+{\text{σ}}^{2}\right)}$, and ${\text{h}}^{2}=\frac{{\text{σ}}_{\text{A}}^{2}}{\left({\text{σ}}_{\text{A}}^{2}+{\text{σ}}_{\text{D}}^{2}+{\text{σ}}^{2}\right)}$, where $\sigma_{\text{A}}^{2}$ and $\sigma_{\text{D}}^{2}$ are the additive and dominance genetic variance components, respectively, and σ^2^ is residual variance. The proportion of genotypic variance among experimental hybrids (hyb) attributed to $\sigma_{\text{GCA}}^{2}$ was calculated as follows (Baker, 1978): ${\text{σ}}_{\text{hyb}/\text{GCA}}^{2}=\frac{2{\text{σ}}_{\text{GCA}}^{2}}{\left(2{\text{σ}}_{\text{GCA}}^{2}+{\text{σ}}_{\text{SCA}}^{2}\right)}$. It indicates the predictability of the hybrids performance based only on the GCA effects. Also, we estimated Pearson’s correlation coefficients between tested traits based on the GCA effects of elite inbred lines of each trait using the R package “stats” (R Core Team, 2022).

#### 2.5.3 Grouping of inbred lines

The 15 elite inbred lines were assigned into putative heterotic groups based on both genetic distance (GD) from SNP markers and estimates of SCA effects for GY across all environments. In the SNP-based GD method, the GD among inbred lines was estimated from 3,083 SNPs markers according to modified Roger’s genetic distance (MRD; Goodman and Stuber, 1983) as follows: $\text{MRD}=\sqrt{\frac{\sum_{\text{i}=1}^{\text{m}}\sum_{\text{j}=1}^{{\text{a}}_{\text{i}}}{\left({\text{p}}_{\text{ij}}-{\text{q}}_{\text{ij}}\right)}^{2}}{2\text{m}}}$, where p_ij_ and q_ij_ are the allele frequencies of the j^th^ allele at the i^th^ locus in the inbred lines in each pair, a_i_ is the number of alleles at the i^th^ marker, and m is the number of marker loci. The MRD was estimated using the R package “adegenet” (Jombart, 2008). Then, we used the MRD matrix to cluster the 15 tropical maize inbred lines based on the Unweighted Pair Group Method using Arithmetic Averages (UPGMA; Sokal and Michener, 1958). In the SCA method, the estimates of SCA effects for GY across all environments were considered as genetic distance among the inbred lines and used to cluster the 15 inbred lines based on UPGMA dendrogram (Sokal and Michener, 1958). Both UPGMA dendrograms were generated using the R package “ape” (Paradis and Schliep, 2019). In both dendrograms, the Mojena method (Mojena, 1977) was used to allocate the inbred lines into heterotic groups. According to this method, the dendrogram must be cut as a function of the mean value of the genetic distance of fusion levels and the standard deviation of the distance values.

Finally, we estimated Pearson’s correlation coefficients between GD among inbred lines and adjusted mean of experimental hybrids, GD and SCA effects, and adjusted mean of hybrids and SCA effects for all tested traits using the R package “stats” (R Core Team, 2022).

## 3 Results

### 3.1 Ranges, means, analysis of variance and correlations among traits

We observed wide ranges among the adjusted mean of hybrids for most traits across all environments, particularly for GY, ranged from 1,840 to 12,500 kg ha^-1^ (range of approximately 10,600 kg ha^-1^; **Table 2** and Supplemental **Supplementary Table 1**). DTP ranged by around 14 days, whereas DTS ranged 17 days. PH ranged from 212.4 to 270.8 cm, and EH ranged from 110 to 160.3 cm. In contrast, BENN and AENN had very small variation, ranging by only three and one nodes, respectively. SD ranged by 6 mm, and LA by 470 cm^2^. For yield components, the EL and ED ranged by 6 cm and 26 cm, respectively; the NKR ranged approximately five kernels rows, and NKE ranged by 360 kernels per ear. For grain morphology, the GA and GP ranged from 69.5 to 112.6 mm^2-^, GP from 33.3 to 42.9 mm, GL ranged from 10.9 to 14.6 mm, and GW from 8.6 to 10.7 mm. Finally, TKW exhibited a range of approximately 170 g.

**Table 2:** Estimates of variance component of block within replication within environment ( $\hat{\sigma }$^2^_b/r/e_), mean squares from the joint analysis, general mean, and ranges (minimum and maximum), and coefficient of variation (CV%) for 18 agronomic traits measured in 105 experimental and five check hybrids of tropical maize across four environments in Brazil.

Source of variation	df	DTP^1/^	DTS	PH	EH	BENN	AENN
${\hat{\sigma }}_{\text{b/r/e}}^{2}$		0.27**^2/^	0.23**	25.6**	13.4**	0.04**	0.04**
Rep/Env	8	1.13	0.25	72.6	118.3*	2.20**	1.89**
Hybrid	109	40.39**^3/^	53.94**	1,542.1**	1,082.9**	2.84**	0.81**
Experimental (Exp)	104	32.82**	43.49**	1,549.8**	1,054.2**	2.88**	0.81**
Check	4	95.00**	99.50**	1,097.0**	1,759.5**	1.73**	1.03**
Exp. *vs* Check	1	1.00	26.00**	2,709.0**	1,251.0**	2.90**	0.10
Environment (Env)	3	2,314.66**	2,479.67**	5,717.3**	4,887.7**	4.47**	2.07**
Exp. x Env	312	3.15**	2.13**	70.9**	69.5**	0.27**	0.19
Residual		1.10	1.11	51.5	51.9	0.21	0.17
CV(%)		1.53	1.55	2.95	5.44	6.03	6.63
Minimum		63.6	62.5	212.4	110.0	6.5	5.7
Mean		68.5	68.1	243.0	132.5	7.5	6.3
Maximum		78.0	80.0	270.8	160.3	9.3	7.0
	df	SD	LA	EL	ED	NKR	NKE
${\hat{\sigma }}_{\text{b/r/e}}^{2}$		0.28**	475.6**	0.09	1.0	0.04*	117.9**
Rep/Env	8	5.13*	3,938.9	2.00	71.5*	0.50	2,575.0
Hybrid	109	11.79**	62,430.4**	13.83**	94.0**	11.29**	31,199.0**
Experimental (Exp)	104	11.94**	64,605.3**	13.88**	93.4**	11.57**	32,547.4**
Check	4	9.50**	26,759.0**	7.25*	79.8	6.75**	3,594.5
Exp. *vs* Check	1	0.00	129.0	27.00**	208.0*	1.00	776.0
Environment (Env)	3	125.00**	4,468,786.3**	339.00**	560.7**	7.67**	237,527.7**
Exp. x Env	312	3.02**	5708.1**	2.61	33.9	0.99**	2,740.5**
Residual		2.33	3832.0	2.51	34.3	0.74	1,997.3
CV(%)		6.90	6.44	9.92	12.29	5.71	8.13
Minimum		19.3	761.4	12.4	36.8	12.3	317.2
Mean		22.1	960.5	16.0	47.6	15.1	549.6
Maximum		25.2	1231.1	18.4	62.6	17.1	677.2
	df	GA	GP	GL	GW	TKW	GY
${\hat{\sigma }}_{\text{b/r/e}}^{2}$		12.8**	1.12**	0.07**	0.03**	52.0**	398,512**
Rep/Env	8	196.8**	16.50**	1.39**	0.70**	542.5	889,413
Hybrid	109	629.5**	27.42**	4.23**	2.07**	1,2032.9**	15,611,009**
Experimental (Exp)	104	592.3**	25.06**	3.91**	2.11**	11,922.0**	15,373,077**
Check	4	185.5**	8.25**	1.68**	0.90**	2,214.8**	10,764,250**
Exp. *vs* Check	1	6,235.0**	348.00**	47.70**	3.10**	63,277.0**	49,693,000**
Environment (Env)	3	701.3**	87.33**	7.17**	3.03**	27,764.3**	108,176,667**
Exp. x Env	312	23.8**	1.47**	0.17**	0.07**	976.6**	2,324,601**
Residual		11.0	0.72	0.08	0.04	598.6	1,667,285
CV(%)		3.47	2.15	2.06	2.00	7.55	13.5
Minimum		69.5	33.3	10.9	8.6	242.0	1,840
Mean		95.6	39.6	13.4	9.6	324.0	9,550
Maximum		112.6	42.9	14.6	10.7	410.8	12,500

^1/^ DTP, days to pollen (days); DTS, days to silking (days); PH, plant height (cm); EH, ear height (cm); BENN, bellow ear node number; AENN, above ear node number; SD, stalk diameter (mm); LA, leaf area (cm^2^); EL, ear length (cm); ED, ear diameter (mm); NKR, number of kernel rows; NKE, number of kernels per ear; GA, grain area (mm^2^); GP, grain perimeter (mm); GL, grain length (mm); GW, grain width (mm); TKW, one thousand kernel weight (g), and grain yield (GY, g ha^-1^). ^2/^**, *significant at P = 0.01 and P = 0.05, respectively, by the likelihood ratio test. ^3/^**, *Significant at P = 0.01 and P = 0.05, respectively, by Wald test.

Joint analysis of variance across four environments showed that experimental hybrids, check hybrids, and environments were highly significant (*P<0.001*) for all 18 tested traits by Wald test (**Table 2**). Also, experimental hybrid x environment interaction was highly significant (*P<0.001*) for most traits, except for AENN, EL and ED. The coefficient of variation (CV) ranged from 1.53% (DTP) to 13.5% (GY) and presented very low values (CV values< 7.0%) for most traits, particularly for plant architecture and grain morphology traits.

**Figure 1:**
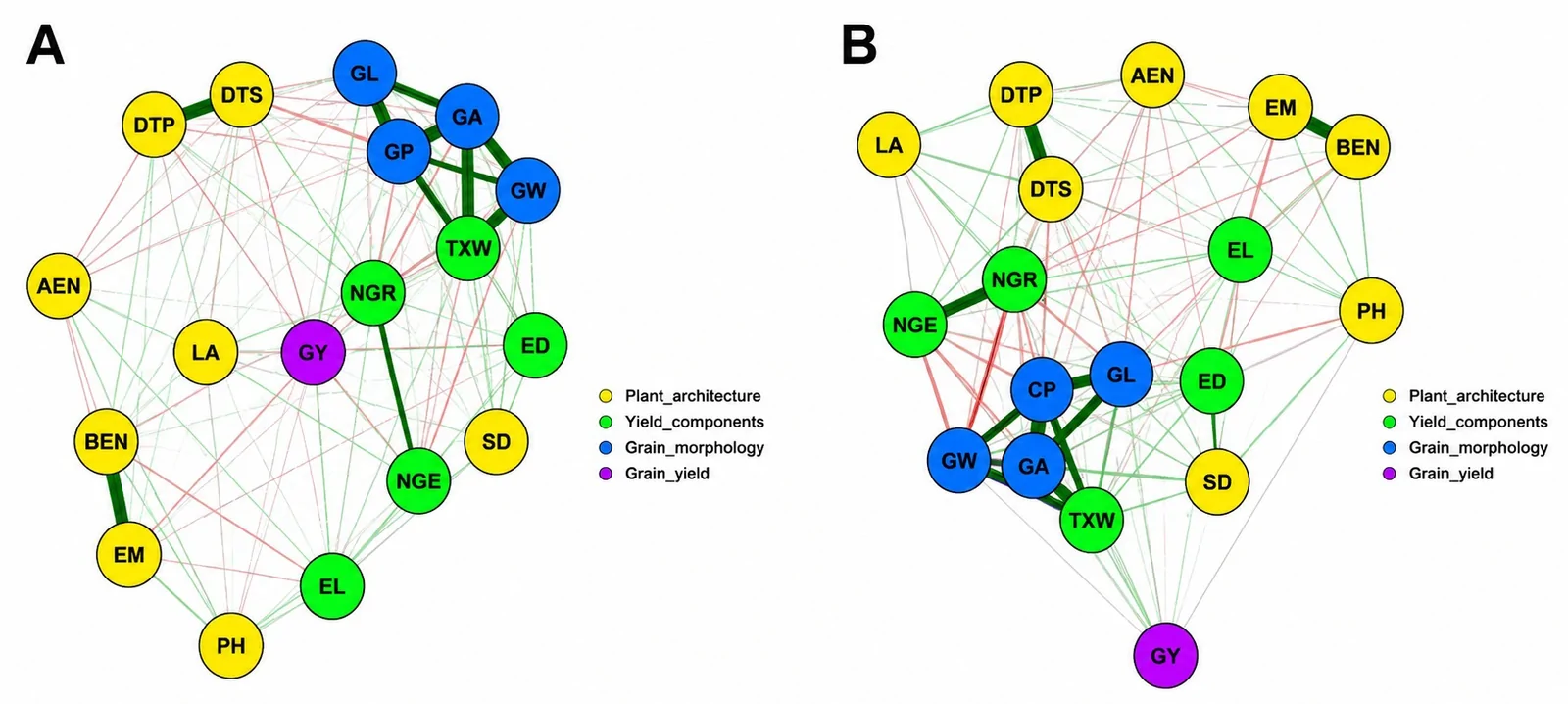
Correlation network between 18 tested agronomic traits based on adjusted means of experimental hybrids **(A)** and based on estimates of general combining effects of 15 tropical maize inbred lines **(B)** across four environments in Brazil. Red and green lines represent negative and positive correlations, respectively. Line width is proportional to the strength of the correlation. DTP, days to pollen (days); DTS, days to silking (days); PH, plant height (cm); EH, ear height (cm); BEN, bellow ear node number; AEN, above ear node number; SD, stalk diameter (mm); LA, leaf area (cm^2^); EL, ear length (cm); ED, ear diameter (mm); NKR, number of kernel rows; NKE, number of kernels per ear; GA, grain area (mm^2^); GP, grain perimeter (mm); GL, grain length (mm); GW, grain width (mm); TKW, one thousand kernel weight (g), and grain yield (GY, kg ha^-1^).

Most Pearson’s correlation coefficients between traits, based on the estimates of adjusted mean of experimental hybrids, were either non-significant (*P>0.10*) or presented low magnitude (**Table 3**, **Figure 1A**). Strongest positive correlations were observed between traits within the same group: DTP and DTS ( $\hat{r}=0.90$) for cycle traits; BENN and EH ( $\hat{r}=0.88$) for plant stature; and for grain morphology: GP and GA ( $\hat{r}=0.97$), GL and GA ( $\hat{r}=0.81$), GL and GP ( $\hat{r}=0.92$), GW and GA ( $\hat{r}=0.88$), and GW and GP ( $\hat{r}=0.75$). Also, strong positive correlations were found between traits from different groups: TKW and GA ( $\hat{r}=0.84$), TKW and GP ( $\hat{r}=0.76$), and TKW and GW ( $\hat{r}=0.79$); and a moderate correlation between two yield components: NKE and NKR ( $\hat{r}=0.71$). Finally, GY exhibited weak negative correlations with DTP ( $\hat{r}=-0.29$) and DTS ( $\hat{r}=-0.34$), but positive correlations with LA ( $\hat{r}=0.21$), EL ( $\hat{r}=0.42$), ED ( $\hat{r}=0.28$), NKE ( $\hat{r}=0.48$), GA ( $\hat{r}=0.33$), GP ( $\hat{r}=0.37$), GL ( $\hat{r}=0.44$) and TKW ( $\hat{r}=0.32$).

**Table 3:** Estimates of Pearson’s correlation coefficients among18 agronomic traits based on estimates of adjusted means of experimental hybrids (above the diagonal) and general combining ability effects (below the diagonal) of tropical maize inbred lines evaluated across four environments in Brazil.

Traits	DTP^1/^	DTS	PH	EH	BENN	AENN	SD	LA	EL	ED	NKR	NKE	GA	GP	GL	GW	TKW	GY
DTP		0.90*^2/^	0.05	0.20*	0.25*	-0.27*	0.17	0.23*	-0.03	-0.04	0.28*	0.03	-0.21*	-0.24*	-0.20*	-0.22*	0.01	-0.29*
DTS	0.90*		0.08	0.19*	0.20*	-0.13	0.14	0.26*	-0.10	-0.03	0.33*	0.01	-0.35*	-0.40*	-0.39*	-0.27*	-0.15	-0.34*
PH	0.20	0.27		0.52*	0.28*	0.31*	0.36*	0.16	0.42*	-0.06	-0.01	0.25*	-0.04	-0.07	-0.12	0.04	0.07	0.18
EH	0.28	0.30	0.47		0.88*	-0.15	0.03	0.05	-0.23*	-0.20*	-0.24*	0.05	0.01	0.03	0.07	-0.01	-0.15	0.13
BENN	0.28	0.24	0.32	0.94*		-0.21*	0.03	0.02	-0.38*	-0.14	-0.24*	-0.06	0.09	0.13	0.17	0.05	-0.12	0.06
AENN	-0.32	-0.13	0.30	-0.23	-0.24		0.04	0.27*	0.22*	-0.10	0.02	-0.05	-0.12	-0.17	-0.23*	0.01	0.04	0.01
SD	0.26	0.24	0.43	-0.02	-0.01	0.01		0.26*	0.35*	0.47*	0.10	0.15	0.37*	0.28*	0.09	0.47*	0.47*	0.04
LA	0.46	0.51	0.01	-0.02	0.03	0.28	0.23		0.12	0.28*	0.31*	0.32*	0.03	0.05	0.10	-0.04	0.10	0.21*
EL	0.20	0.12	0.38	-0.47	-0.50	0.27	0.44	-0.10		0.19	0.29*	0.53*	0.05	0.03	0.01	0.01	0.29*	0.42*
ED	0.09	0.07	-0.26	-0.36	-0.22	-0.22	0.66*	0.28	0.13		0.32*	0.32*	0.39*	0.41*	0.35*	0.29*	0.35*	0.28*
NKR	0.44	0.48	-0.09	-0.32	-0.30	0.03	0.09	0.31	0.41	0.26		0.71*	-0.51*	-0.44*	-0.29*	-0.61*	-0.31*	0.12
NKE	0.42	0.39	0.04	-0.13	-0.09	-0.18	0.10	0.15	0.39	0.25	0.85*		-0.25*	-0.15	0.02	-0.45*	-0.25*	0.48*
GA	-0.19	-0.35	-0.14	-0.04	0.10	-0.22	0.43	-0.07	-0.09	0.50	-0.60*	-0.54*		0.97*	0.81*	0.88*	0.84*	0.33*
GP	-0.19	-0.38	-0.22	-0.02	0.14	-0.31	0.31	-0.08	-0.17	0.49	-0.57*	-0.47	0.97*		0.92*	0.75*	0.76*	0.37*
GL	-0.10	-0.35	-0.32	0.02	0.20	-0.43	0.04	-0.05	-0.24	0.34	-0.47	-0.33	0.82*	0.93*		0.46*	0.58*	0.44*
GW	-0.28	-0.32	0.03	-0.02	0.05	-0.01	0.59*	-0.07	-0.04	0.48	-0.63*	-0.62*	0.91*	0.80*	0.53*		0.79*	0.11
TKW	0.03	-0.15	-0.02	-0.24	-0.13	-0.04	0.58*	0.04	0.32	0.48	-0.34	-0.40	0.87*	0.79*	0.62*	0.82*		0.32*
GY	-0.04	-0.11	-0.19	-0.02	0.09	-0.06	-0.07	-0.17	0.11	0.15	0.03	0.11	0.30	0.31	0.39	0.13	0.35	

^1/^ DTP, days to pollen (days); DTS, days to silking (days); PH, plant height (cm); EH, ear height (cm); BENN, bellow ear node number; AENN, above ear node number; SD, stalk diameter (mm); LA, leaf area (cm^2^); EL, ear length (cm); ED, ear diameter (mm); NKR, number of kernel rows; NKE, number of kernels per ear; GA, grain area (mm^2^); GP, grain perimeter (mm); GL, grain length (mm); GW, grain width (mm); TKW, one thousand kernel weight (g), and grain yield (GY, kg ha^-1^). Pearson correlation coefficients lower than -0.60 or higher than 0.60 are highlighted in bold.^2/^* significant at P = 0.05 by t test.

### 3.2 Variance components and heritability estimates

Variance components of GCA and SCA were significant (*P< 0.05*) by likelihood ratio test for all traits across environments (**Table 4**). Regarding interactions, the variance components of GCA x environment interaction were significant (*P<0.05*) for almost all traits, except SD and ED, whereas variance component of SCA x environment interaction was significant (*P<0.05*) only for LA and GY. Estimates of additive genetic variance component ( ${\hat{\sigma }}_{\text{A}}^{2}$) were substantially higher than dominance genetic variance ( ${\hat{\sigma }}_{\text{D}}^{2}$) for all plant architecture and grain morphology traits, as well as for the yield components ED, NKR and TKW. Conversely, the ${\hat{\sigma }}_{\text{D}}^{2}$ exceeded ${\hat{\sigma }}_{\text{A}}^{2}$ for the yield components NKE and EL, and GY by approximately 15%, 25%, and 180%, respectively.

**Table 4:** Estimates of variance component of general (σ^2^_GCA_) and specific (σ^2^_SCA_) combining ability, GCA x environment (σ^2^_GCAxE_) and SCA x environment (σ^2^_SCAxE_) interaction, residual variance (σ^2^), additive (σ^2^_A_) and dominance (σ^2^_D_) genetic variance components, narrow-sense (h^2^) and broad-sense heritability (H^2^), and proportion of genotypic variance estimates of experimental hybrids attributed to GCA (σ^2^_hyb/GCA_) for 18 agronomic traits measured in 105 experimental hybrids of tropical maize across four environments in Brazil.

Parameters	DTP^1/^	DTS	PH	EH	BENN	AENN
${\hat{\sigma }}_{\text{GCA}}^{2}$	0.60**^2/^	0.77**	33.60**	23.30**	0.06**	0.01**
${\hat{\sigma }}_{\text{SCA}}^{2}$	0.54**	0.79**	25.05**	11.10**	0.01**	0.01**
${\hat{\sigma }}_{\text{GCAxE}}^{2}$	0.09**	0.10**	2.09**	2.16**	4.17x10^-3^**	1.66x10^-3^**
${\hat{\sigma }}_{\text{SCAxE}}^{2}$	0.08	0.11	0.35	~ 0	~ 0	~ 0
${\hat{\sigma }}^{2}$	0.40	0.38	19.23	17.91	0.08	0.06
${\hat{\sigma }}_{\text{A}}^{2}$	1.21	1.54	67.20	46.60	0.12	0.02
${\hat{\sigma }}_{\text{D}}^{2}$	0.54	0.79	25.05	11.10	0.01	0.01
${\hat{H}}^{2}$	0.81	0.86	0.83	0.76	0.60	0.37
${\hat{h}}^{2}$	0.56	0.57	0.60	0.62	0.56	0.24
${\hat{\sigma }}_{\text{hyb/GCA}}^{2}$	0.69	0.66	0.73	0.81	0.93	0.66
	SD	LA	EL	ED	NKR	NKE
${\hat{\sigma }}_{\text{GCA}}^{2}$	0.22**	1,411.76**	0.15**	1.03**	0.20**	492.52**
${\hat{\sigma }}_{\text{SCA}}^{2}$	0.12**	840.15**	0.39**	1.20*	0.15**	1,118.80**
${\hat{\sigma }}_{\text{GCAxE}}^{2}$	0.01	116.83**	0.02*	0.04	0.02**	36.08**
${\hat{\sigma }}_{\text{SCAxE}}^{2}$	0.01	288.10*	~ 0	3.06	0.03	~ 0
${\hat{\sigma }}^{2}$	1.00	1,231.14	0.85	9.33	0.25	844.99
${\hat{\sigma }}_{\text{A}}^{2}$	0.44	2,823.51	0.31	2.06	0.41	985.05
${\hat{\sigma }}_{\text{D}}^{2}$	0.12	840.15	0.39	1.20	0.15	1,118.80
${\hat{H}}^{2}$	0.36	0.75	0.45	0.26	0.69	0.71
${\hat{h}}^{2}$	0.28	0.58	0.20	0.16	0.51	0.33
${\hat{\sigma }}_{\text{hyb/GCA}}^{2}$	0.78	0.77	0.44	0.63	0.73	0.47
	GA	GP	GL	GW	TKW	GY
${\hat{\sigma }}_{\text{GCA}}^{2}$	10.77**	0.42**	0.07**	0.05**	249.67**	126,945.2**
${\hat{\sigma }}_{\text{SCA}}^{2}$	7.27**	0.33**	0.08**	0.02**	160.73**	704,283.6**
${\hat{\sigma }}_{\text{GCAxE}}^{2}$	0.99**	0.06**	0.01**	2.89x10^-3^**	26.24**	37,591.4**
${\hat{\sigma }}_{\text{SCAxE}}^{2}$	0.33	0.04	~ 0	~ 0	30.42	140,466.9*
${\hat{\sigma }}^{2}$	5.07	0.34	0.03	0.02	226.31	589,908.5
${\hat{\sigma }}_{\text{A}}^{2}$	21.54	0.84	0.15	0.09	499.35	253,890.5
${\hat{\sigma }}_{\text{D}}^{2}$	7.27	0.33	0.08	0.02	160.73	704,283.5
${\hat{H}}^{2}$	0.85	0.78	0.87	0.87	0.74	0.62
${\hat{h}}^{2}$	0.64	0.56	0.58	0.72	0.56	0.16
${\hat{\sigma }}_{\text{hyb/GCA}}^{2}$	0.75	0.72	0.66	0.83	0.76	0.26

^1/^ DTP, days to pollen (days); DTS, days to silking (days); PH, plant height (cm); EH, ear height (cm); BENN, bellow ear node number; AENN, above ear node number; SD, stalk diameter (mm); LA, leaf area (cm^2^); EL, ear length (cm); ED, ear diameter (mm); NKR, number of kernel rows; NKE, number of kernels per ear; GA, grain area (mm^2^); GP, grain perimeter (mm); GL, grain length (mm); GW, grain width (mm); TKW, one thousand kernel weight (g), and grain yield (GY, kg ha^-1^). ^2/^**, * significant at P = 0.01 and P = 0.05, respectively, by the likelihood ratio test.

Estimates of broad-sense heritability ( ${\hat{H}}^{2}$) ranged from 0.26 (ED) to 0.87 (GL and GW), and most traits exhibited high ${\hat{H}}^{2}$ values (>0.70), particularly for cycle, plant stature and grain morphology traits. Estimates of narrow-sense heritability ( ${\hat{h}}^{2}$) ranged from 0.16 (ED and GY) to 0.72 (GW). Grain morphology, cycle and plant stature (PH and EH) traits, along with BENN, LA, NRK and TKW, showed intermediate ${\hat{h}}^{2}$ values (>0.50), whereas AEEN ( ${\hat{h}}^{2}=0.24$), SD ( ${\hat{h}}^{2}=0.28$), and yield components EL ( ${\hat{h}}^{2}=0.20$), ED ( ${\hat{h}}^{2}=0.16$) and NKE ( ${\hat{h}}^{2}=0.36$), as well as GY ( ${\hat{h}}^{2}=0.16$), exhibited ${\hat{h}}^{2}$ values (<0.40). Notably, the ${\hat{h}}^{2}$ for GY was 75% lower than its ${\hat{H}}^{2}$ values, representing the largest difference between broad- and narrow-sense heritability among tested traits. The proportion of genotypic variance estimates of hybrids attributed to GCA ( ${\hat{\sigma }}_{\text{hyb/GCA}}^{2}$) ranged from 0.26 (GY) to 0.93 (BENN), and most traits had ${\hat{\sigma }}_{\text{hyb/GCA}}^{2}$ values greater than 0.60, except for EL (0.44), NKE (0.47) and GY (0.26).

### 3.3 GCA effects

Although most Pearson’s correlation coefficients based on GCA effects between traits were either non-significant (*P>0.05*) or of low magnitude, we observed moderate-to-strong positive association (>0.60) for some trait pairs (**Table 3**, **Figure 1B**). Similarly, correlations based on adjusted means, we found strong positive associations within trait groups: DTP and DTS ( ${\hat{r}}^{2}=0.90$) for cycle traits; EH and BENN ( ${\hat{r}}^{2}=0.94$) for plant stature; NKR and NKE ( ${\hat{r}}^{2}=0.85$) for yield components; GA and GP ( ${\hat{r}}^{2}=0.97$), GP and GL ( ${\hat{r}}^{2}=0.93$), GA and GW ( ${\hat{r}}^{2}=0.91$), GA and GL ( ${\hat{r}}^{2}=0.82$), GP and GW ( ${\hat{r}}^{2}=0.80$) for grain morphology. Strong positive correlations were also observed between traits from different groups, including GA and TKW ( ${\hat{r}}^{2}=0.87$), GW and TKW ( ${\hat{r}}^{2}=0.82$), and GP and TKW ( ${\hat{r}}^{2}=0.79$). Also, moderate positive correlations were observed between SD and ED ( ${\hat{r}}^{2}=0.66$), GL and TKW ( ${\hat{r}}^{2}=0.62$), SD and GW ( ${\hat{r}}^{2}=0.59$), and SD and TKW ( ${\hat{r}}^{2}=0.58$). In contrast, negative moderate correlations were observed between yield components and morphology grain traits: GW and NKR ( ${\hat{r}}^{2}=-0.63$), NKE and GW ( ${\hat{r}}^{2}=-0.62$), NKR and GA ( ${\hat{r}}^{2}=-0.60$), NKR and GP ( ${\hat{r}}^{2}=-0.57$), and NKE and GA ( ${\hat{r}}^{2}=-0.54$). Finally, the GY exhibited no significant (*P< 0.10*) correlation with any tested trait.

Regarding GCA effects, the inbred lines VML004, VML016, VML062, VML081, VML083, VML131 and VML157 had positive GCA effects for GY (**Table 5**). Most of these lines also presented negative GCA effects for at least one cycle trait and one plant stature trait. Notably, VML131 had the highest GCA effects for GY (1,187 kg ha^-1^), along with negative GCA effects for DTP (-0.5 day), DTS (-0.7 day), PH (-7 cm), EH (-4 cm); and positive GCA effects for TKW (41 g) and almost all grain morphology traits. The VML062 also showed favorable performance for GY (120 kg ha^-1^), cycle (GCA=-0.9 and -0.4 day for DTP and DTS, respectively), and plant stature (GCA=-6 and -7 cm, for PH and EH, respectively). Similarly, VML016 had a positive GCA effect for GY (85 kg ha^-1^) associated with negative GCA for EH (-12 cm). The VML083 also showed a positive GCA effect for GY (256 kg ha^-1^) while reducing the PH (-9 cm); and the line VML081 had a positive GCA effect for GY (45 kg ha^-1^) along with desirable GCA for DTP (-2 days) and DTS (-2 days). Although VML004 exhibited a high GCA effect for GY (535 kg ha^-1^), it had undesirable GCA effects for EH (12 cm) and PH (5 cm). Similarly, VML157 presented a high GCA effect for GY (226 kg ha^-1^), but also showed GCA effects for increasing cycle duration (DTP = 2 days; DTS = 3 days) and PH (4 cm). In contrast, VML105 had very low GCA values for PH (-13 cm) and EH (-4 cm), but negative GCA for GY (-264). Overall, VML016, VML062, VML081, VML083 and VML131 are promising tropical maize inbred lines for simultaneously improving GY and reducing both cycle and plant stature in our breeding program.

**Table 5:** Estimates of general combining ability effects of 15 tropical maize inbred lines for 18 agronomic traits across four environments in Brazil.

Inbred lines	DTP^1/^	DTS	PH	EH	BENN	AENN	SD	LA	EL
VML131	-0.54	-0.74	-6.83	-4.08	-0.22	-0.02	0.21	7.22	0.07
VML004	0.37	-0.13	4.75	12.43	0.83	-0.12	0.05	-45.16	-0.11
VML083	0.21	-0.83	-8.83	-0.66	0.12	-0.03	-0.70	-17.13	-0.13
VML157	2.04	2.87	4.47	0.87	0.18	0.12	0.98	103.45	0.18
VML062	-0.87	-0.36	-5.76	-7.47	-0.32	0.01	-0.56	-32.27	0.28
VML016	-0.17	-0.94	4.24	-12.13	-0.60	0.12	0.20	-39.26	1.25
VML081	-2.54	-2.13	0.03	2.84	0.10	0.22	-1.23	-16.40	-0.98
VML134	1.48	1.88	11.97	8.79	0.17	-0.09	-0.31	2.89	0.26
VML115	-0.64	-1.39	-4.82	-1.08	-0.07	-0.31	0.51	-60.35	-0.27
VML066	-0.57	0.00	1.06	0.85	-0.03	-0.12	0.50	-47.12	-0.19
VML154	1.60	1.79	5.58	3.77	0.09	-0.08	-0.13	0.39	0.06
VML105	0.32	0.94	-13.59	-4.28	-0.26	0.01	-0.68	1.35	-0.29
VML176	-1.26	-1.00	11.55	-2.62	-0.25	0.33	0.95	6.41	0.79
VML140	0.37	-0.10	-7.80	-6.03	-0.24	-0.07	-0.05	101.96	-0.30
VML055	0.18	0.15	4.00	8.81	0.51	0.03	0.24	34.02	-0.65
Minimum	-2.54	-2.13	-13.59	-12.13	-0.60	-0.31	-1.23	-60.35	-0.98
Maximum	2.04	2.87	11.97	12.43	0.83	0.33	0.98	103.45	1.25
	ED	NKR	NKE	GA	GP	GL	GW	TKW	GY
VML131	1.20	-0.48	-17.70	8.69	1.48	0.51	0.49	41.11	1,186.89
VML004	0.09	-0.11	22.46	1.64	0.52	0.33	-0.01	-5.70	535.02
VML083	-1.51	0.27	11.16	-2.14	-0.32	0.03	-0.29	-5.25	256.41
VML157	2.41	1.15	25.68	-0.21	-0.08	-0.10	0.00	11.50	225.77
VML062	-0.08	0.47	17.35	-3.19	-0.27	0.04	-0.26	-10.69	120.53
VML016	0.21	-0.11	-2.52	3.19	0.59	0.19	0.10	27.53	84.67
VML081	-2.04	-0.70	-33.43	-3.43	-0.45	-0.08	-0.18	-31.59	44.79
VML134	-1.97	0.37	24.96	-6.88	-1.26	-0.30	-0.49	-23.96	-107.96
VML115	1.70	-0.26	-18.28	7.55	1.67	0.57	0.37	22.78	-192.89
VML066	1.34	-0.16	16.68	-0.44	-0.29	-0.35	0.16	-15.60	-218.38
VML154	-1.89	0.06	-2.62	-6.13	-1.32	-0.46	-0.28	-12.82	-250.65
VML105	-0.13	0.48	-6.61	-4.32	-1.04	-0.41	-0.19	-16.31	-264.18
VML176	-0.23	0.21	-1.99	-2.48	-1.03	-0.69	0.19	3.45	-437.20
VML140	1.57	0.50	29.79	-1.15	0.08	0.21	-0.21	-12.59	-489.43
VML055	-0.68	-1.68	-64.95	9.29	1.72	0.52	0.60	28.13	-493.37
Minimum	-2.04	-1.68	-64.95	-6.88	-1.32	-0.69	-0.49	-31.59	-493.37
Maximum	2.41	1.15	29.79	9.29	1.72	0.57	0.60	41.11	1,186.89

^1/^ DTP, days to pollen (days); DTS, days to silking (days); PH, plant height (cm); EH, ear height (cm); BENN, bellow ear node number; AENN, above ear node number; SD, stalk diameter (mm); LA, leaf area (cm^2^); EL, ear length (cm); ED, ear diameter (mm); NKR, number of kernel rows; NKE, number of kernels per ear; GA, grain area (mm^2^); GP, grain perimeter (mm); GL, grain length (mm); GW, grain width (mm); TKW, one thousand kernel weight (g), and grain yield (GY, kg ha^-1^).

### 3.4 Correlations between hybrid performance, SCA effects and GD among lines

Pearson’s correlation coefficients between the adjusted mean of experimental hybrids and SCA effects were positive and ranged from 0.28 (BENN) to 0.84 (GY, **Table 6**). Hybrid performance showed moderate correlation (<0.60) with SCA effects for almost all traits, except for EL ( ${\hat{r}}^{2}=0.69$), ED ( ${\hat{r}}^{2}=0.65$), NKE ( ${\hat{r}}^{2}=0.73$) and GY ( ${\hat{r}}^{2}=0.84$). Genetic distances (GD) among inbred lines were moderately negatively correlated with hybrid adjusted mean for DTP ( ${\hat{r}}^{2}=-0.61$) and DTS ( ${\hat{r}}^{2}=-0.66$), and moderately positively correlated with hybrid performance for grain morphology traits (>0.40) and GY ( ${\hat{r}}^{2}=-0.64$). For other traits, GD showed low (<0.35) or no correlation with hybrid performance. Similarly, GD was negatively correlated with SCA effects for DTP ( ${\hat{r}}^{2}=-0.69$) and DTS ( ${\hat{r}}^{2}=-0.72$), and positively correlated with SCA effects for GY ( ${\hat{r}}^{2}=-0.70$). Moreover, GD among inbred lines showed moderate and positive correlation with SCA effects for most other traits, except for BENN and GW. Overall, GD among inbred lines presented stronger correlation with SCA effects than with hybrid performance, but weaker correlation than association between hybrid performance and SCA effects.

**Table 6:** Estimates of Pearson’s correlation coefficients between adjusted means of experimental hybrids and SCA effects (r_hybrids_SCA_), adjusted mean of experimental hybrids and modified Roger’s genetic distances (r_hybrids_GD_), and SCA effects and modified Roger’s genetic distances (r_SCA_GD_).

Trait	${\hat{r}}_{\text{hybrids\_SCA}}$	${\hat{r}}_{\text{hybrids\_GD}}$	${\hat{r}}_{\text{SCA\_GD}}$
DTP^1/^	0.46**^2/^	-0.61**	**-**0.69**
DTS	0.46**	-0.66**	-0.72**
PH	0.51**	0.26**	0.58**
EH	0.38**	0.05	0.32**
BENN	0.28**	~ 0	-0.05
AENN	0.55**	0.21*	0.29**
SD	0.50**	0.24*	0.24*
LA	0.49**	0.20*	0.70**
EL	0.69**	0.34**	0.57**
ED	0.65**	0.38**	0.49**
NKR	0.45**	-0.11	0.32**
NKE	0.73**	0.32**	0.65**
GA	0.34**	0.53**	0.53**
GP	0.39**	0.56**	0.63**
GL	0.46**	0.51**	0.63**
GW	0.30**	0.41**	0.16
TKW	0.42**	0.39**	0.37**
GY	0.84**	0.64**	0.70**

^1/^ DTP, days to pollen (days); DTS, days to silking (days); PH, plant height (cm); EH, ear height (cm); BENN, bellow ear node number; AENN, above ear node number; SD, stalk diameter (mm); LA, leaf area (cm^2^); EL, ear length (cm); ED, ear diameter (mm); NKR, number of kernel rows; NKE, number of kernels per ear; GA, grain area (mm^2^); GP, grain perimeter (mm); GL, grain length (mm); GW, grain width (mm); TKW, one thousand kernel weight (g), and grain yield (GY, kg ha^-1^). ^2/^**, * significant at P = 0.01 and P = 0.05 by t test, respectively.

### 3.5 Grouping of inbred lines

**Figure 2:**
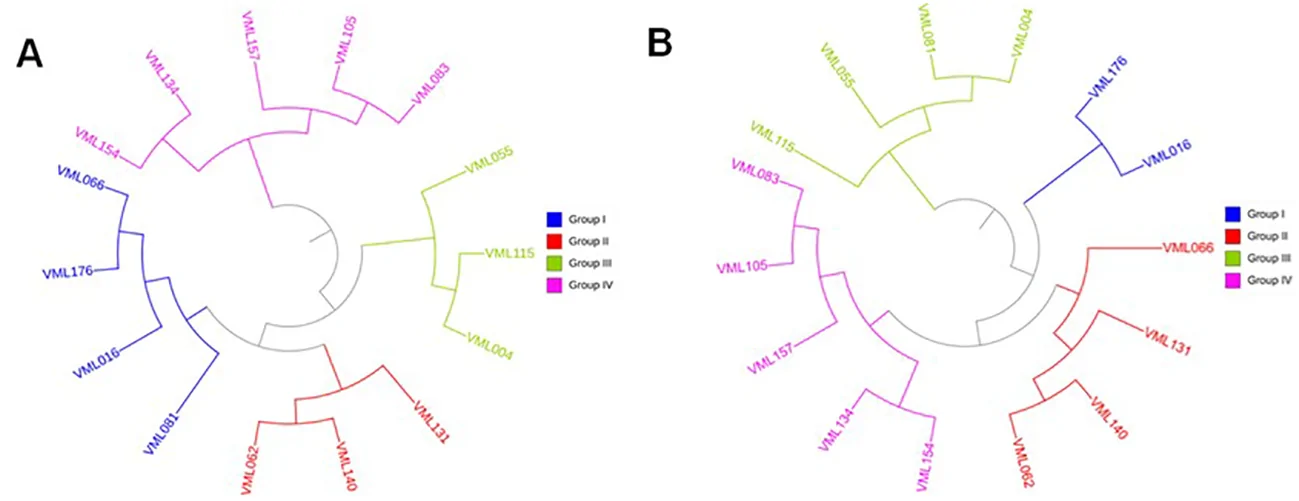
Dendrograms from unweighted pair-group method of arithmetic clustering for 15 tropical maize inbred lines using estimates of specific combining ability effects for grain yield across four environments in Brazil **(A)** and using Roger’s modified genetic distances calculated from 3,083 SNP markers **(B)**.

The estimates of SCA effects for GY ranged from -5,214.67 kg ha^-1^ (VML134 and VML154) to 1,881.53 kg ha^-1^ (VML081 and VML134; **Table 7** and Supplemental **Supplementary Table 2**). Using Mojena’s method (1977), the UPGMA dendrogram grouped the 15 tropical maize inbred lines into four groups based on values of SCA effects for GY (**Figure 2**). Group I consisted of inbred lines VML016, VML066, VML081 and VML176; Group II consisted of VML062, VML131 and VML140; Group III consisted of VML004, VML055 and VML115; and Group IV consisted of lines VML083, VML105, VML134, VML154 and VML157. The VML134 and VML154 allocated into group IV, were the most similar lines, whereas VML081 and VML134 were allocated into groups I and IV, respectively, and were the most divergent inbred lines in terms of SCA effects for GY.

**Table 7:** Estimates of SCA effects for grain yield (above the diagonal) and modified Roger’s genetic distances (bellow the diagonal) among 15 tropical maize inbred lines across four environments in Brazil.

Lines	VML004	VML016	VML055	VML062	VML066	VML081	VML083	VML105	VML115	VML131	VML134	VML140	VML154	VML157	VML176
VML004		-117.85	-234.33	211.73	558.09	-609.61	49.96	491.06	-941.10	58.10	**1,518.94**	25.83	**1,215.67**	925.89	-330.69
VML016	1.2028		-391.07	727.39	-74.74	27.56	245.45	727.05	327.21	161.88	498.03	-162.61	563.26	**1,119.02**	-1,138.47
VML055	1.1915	**1.2435**		209.08	206.15	148.27	481.34	1,037.38	-497.58	-226.14	673.97	136.06	661.31	-292.22	619.62
VML062	1.1652	1.1882	1.2049		5.15	-364.36	311.84	304.63	32.58	-361.14	**1,255.86**	-1,434.95	1,026.83	771.42	347.34
VML066	**1.2295** ^1/^	1.1819	1.2093	1.1778		-812.55	172.87	599.34	344.52	-394.79	956.41	221.48	886.24	618.44	-1,369.27
VML081	1.0885	1.2172	1.1776	1.1846	1.2104		-57.83	490.50	362.16	290.68	**1,881.53**	-338.53	**1,720.53**	-149.98	-63.16
VML083	1.2133	1.2133	**1.2464**	1.2128	1.1872	1.2169		-1,481.10	-294.46	256.25	755.42	**1,674.56**	940.97	-149.24	389.10
VML105	1.2151	1.1821	1.2158	1.1648	1.2132	1.1902	0.9764		428.05	152.11	-384.23	396.32	-279.25	-796.83	**1,451.71**
VML115	1.1934	1.2083	1.2125	1.2059	1.1818	1.2049	1.1854	1.1972		939.88	175.05	759.99	506.86	249.04	593.67
VML131	1.2084	1.2187	**1.2220**	1.1405	1.1472	1.2032	**1.2209**	1.1723	**1.2220**		**1,401.22**	-766.05	**1,259.85**	292.77	-2.46
VML134	1.2001	1.1916	1.2038	1.1757	1.2131	1.1822	1.1205	0.9965	1.2196	**1.2255**		1,016.17	-5,214.67	-291.08	567.44
VML140	1.1543	1.1908	1.2111	0.9128	1.1885	1.2006	1.2040	1.1444	1.1813	1.1495	1.1652		801.82	-369.10	535.01
VML154	1.2010	1.1936	1.2048	1.1722	1.2129	1.1751	1.1133	1.0055	1.2184	1.2114	0.3304	1.1594		-238.35	721.50
VML157	1.2059	1.1600	1.2172	1.1906	1.1733	1.1629	1.0943	1.0106	1.2096	1.1775	1.1188	1.1724	1.1104		840.71
VML176	1.1882	1.1852	1.2144	1.1897	1.2095	1.2016	1.2187	1.2112	**1.2376**	**1.2308**	1.1971	1.2032	1.1942	**1.2285**	

^1/^ The ten highest estimates of SCA effects and modified Roger’s genetic distances are highlighted in bold.

Regarding the SNP-based GD method, the mean of Roger’s modified genetic distance among the 15 inbred lines was 1.1734, ranging from 0.3304 (VML134 and VML154) to 1.2464 (VML055 and VML083; **Table 7**). The UPGMA dendrogram grouped the 15 inbred lines into four groups based on Mojena’s method (1977; **Figure 2**). Group I consisted of two lines, VML016 and VML176; Group II consisted of lines VML062, VML066, VML131 and VML140; Group III of lines VML004, VML055, VML081 and VML115; and Group IV consisted of lines VML083, VML105, VML134, VML154 and VML157.

As we observed a strong and positive correlation between GD among inbreed lines and their SCA effects for GY ( ${\hat{r}}^{2}=-0.70$), there was a close correspondence between lines grouping by both methods in terms of number of groups and placement of inbred lines. Only the inbred lines VML066 and VML081 were not allocated into same group by the two approaches. The lines VML083, VML105, VML134, VML154 and VML157, and VML016 and VML176 were grouped together by the two methods. Similarly, the sets of three lines VML062, VML131 and VML140; and VML004, VML055 and VML115 were also consistently placed into the same groups by both approaches.

### 3.6 Hybrid performance

Two out of the 15 highest-yielding tropical maize hybrids evaluated in our study outperformed the best check, BM709PRO2 (**Table 8**). The hybrid VML131/VML134 (12,545 kg ha^-1^) out-yielded the best commercial check, BM709PRO2 (11,794 kg ha^-1^), by approximately 6%, whereas the hybrid VML004/VML134 (11,989 kg ha^-1^) showed a slightly higher mean yield (around 200 kg ha^-1^) than the best check. In addition to these two hybrids, eleven other experimental hybrids had mean yields higher the second highest-yielding check, DKB390PRO3 (10,887 kg ha^-1^). Overall, the field performance of the highest-yielding experimental hybrids was comparable to that of the commercial checks for plant stature, yield components, and grain morphology, except of the hybrid VML004/VML134. Although VML004/VML134 was the second highest-yielding hybrid, it had very high mean PH (265.76 cm) and EH (155.20 cm) compared with the commercial hybrids used in our study. Conversely, the highest-yielding hybrid, VML131/VML134, had lower mean values for architecture traits and days to flowering than the best commercial check combined with mean yield components higher than best check. Additionally, the hybrid VML081/VML131 was the earliest (around 65 days for days to flowering), whereas the hybrid VML083/VML140 was the shortest (226.74 cm) among the highest-yielding hybrids and also presented a low ear insertion height (EH = 130.37 cm).

**Table 8:** Adjusted means for 18 agronomic traits of the 15 (~14%) highest-yielding tropical maize hybrids across four environments in Brazil.

Hybrid	DTP^1/^	DTS	PH	EH	BENN	AENN	SD	LA	EL
VML131/VML134	67.81	67.84	245.53	134.37	7.31	6.13	21.91	1,001.28	17.45
VML004/VML134	69.59	69.14	265.76	155.20	8.41	6.04	21.74	979.03	16.63
BM709PRO2	68.61	69.91	249.41	140.98	7.83	6.56	22.33	980.70	17.46
VML081/VML134	66.67	67.01	262.20	151.62	8.09	6.14	21.83	985.67	15.89
VML115/VML131	68.59	67.11	229.79	131.07	7.32	5.78	22.24	920.07	16.67
VML004/VML157	71.62	71.24	254.65	148.26	8.60	6.28	23.53	1,008.57	15.70
VML004/VML131	69.32	68.19	245.85	143.14	8.12	6.17	21.92	914.11	16.59
VML131/VML157	69.51	69.70	243.19	132.08	7.55	6.32	23.56	1,045.38	15.81
VML131/VML154	68.41	67.52	242.30	129.76	7.31	6.17	22.38	988.34	16.36
VML083/VML131	67.84	66.37	230.81	129.76	7.43	6.20	22.51	959.15	16.62
VML083/VML140	69.09	66.92	226.74	130.37	7.42	6.09	20.84	1,058.58	15.97
VML081/VML131	65.48	65.65	237.58	133.58	7.47	6.58	20.32	958.95	15.13
VML016/VML157	70.08	70.24	261.43	126.01	7.08	6.54	23.35	990.97	17.87
VML016/VML131	67.70	66.63	236.82	117.30	6.85	6.21	21.79	914.83	16.99
DKB390PRO3	71.15	66.83	236.23	141.03	7.62	5.74	21.74	1,008.68	15.77
Hybrid	ED	NKR	NKE	GA	GP	GL	GW	TKW	GY
VML131/VML134	48.29	15.36	638.4	96.51	39.83	13.73	9.44	329.2	12,545
VML004/VML134	45.50	15.06	618.3	92.45	39.06	13.66	9.12	296.6	11,989
BM709PRO2	47.82	14.48	519.1	106.28	42.37	14.41	9.86	364.8	11,794
VML081/VML134	42.97	14.63	559.6	86.24	37.93	13.05	8.96	279.9	11,584
VML115/VML131	49.29	13.54	494.4	111.59	42.20	14.12	10.67	410.8	11,567
VML004/VML157	51.11	16.20	595.9	99.16	40.31	13.67	9.71	342.6	11,343
VML004/VML131	49.46	14.51	545.1	107.90	41.85	14.29	10.21	368.9	11,297
VML131/VML157	50.67	15.16	537.1	102.21	40.50	13.58	10.02	380.3	11,266
VML131/VML154	48.03	15.02	540.6	98.10	40.04	13.54	9.74	358.5	11,234
VML083/VML131	47.82	15.37	576.1	99.35	40.41	13.86	9.62	342.9	11,101
VML083/VML140	47.11	15.61	584.6	94.52	39.89	13.81	9.17	331.3	11,045
VML081/VML131	47.29	13.79	504.7	98.64	40.09	13.55	9.90	320.2	10,991
VML016/VML157	50.12	16.74	604.1	97.37	40.01	13.59	9.54	370.1	10,978
VML016/VML131	47.99	14.89	529.0	106.04	41.14	14.05	10.04	370.7	10,894
DKB390PRO3	52.84	16.51	567.1	101.88	40.48	13.56	9.87	348.5	10,887

^1/^ DTP, days to pollen (days); DTS, days to silking (days); PH, plant height (cm); EH, ear height (cm); BENN, bellow ear node number; AENN, above ear node number; SD, stalk diameter (mm); LA, leaf area (cm^2^); EL, ear length (cm); ED, ear diameter (mm); NKR, number of kernel rows; NKE, number of kernels per ear; GA, grain area (mm^2^); GP, grain perimeter (mm); GL, grain length (mm); GW, grain width (mm); TKW, one thousand kernel weight (g), and grain yield (GY, kg ha^-1^).

## 4 Discussion

The significant and large genotypic variation observed among the experimental hybrids of tropical maize for plant architecture, grain morphology, yield components and grain yield -associated with both very low CV values for all traits and high ${\hat{H}}^{2}$ values for most tested traits across environments - suggests that the selection for improving those traits will result in substantial genetic progress in this set of maize hybrids under tropical environments (Prasanna et al., 2021; Masuka et al., 2017; De La Fuente et al., 2013; Hallauer et al., 2010). The presence of hybrid x environment interaction for GY and most other traits indicates that hybrids respond to environmental variations and the need for extensive testing of tropical maize hybrids across multiple environments to avoid incorrect cultivar recommendations (Yan et al., 2016; Ceccarelli et al., 2015; Stojaković et al., 2015). According to Uberti et al. (2023), the evaluation and the selection of high-yielding and stable maize hybrids across tropical environments in Brazil must be performed over at least two growing seasons for each of the target environment. Moreover, the selection for increasing GY in our set of hybrids can be done along with simultaneous desirable genetic gains for cycle and plant stature since GY was moderately negatively associated with flowering time and showed no correlation with plant architecture traits. The yield performance of hybrids was also positively associated with number of kernels per plant and kernel size and weight, highlighting the importance of considering the yield components and grain morphology traits in the development of high-yielding tropical maize hybrids (Li et al., 2021).

The greater ${\hat{\sigma }}_{\text{A}}^{2}$ than ${\hat{\sigma }}_{\text{D}}^{2}$ values associated with high ${\hat{\sigma }}_{\text{hyb/GCA}}^{2}$ values for flowering time, plant architecture, kernel size and weight, and the yield components ED and NKR, indicate that the additive genetic action predominantly governed the inheritance of these traits in tropical maize. Similarly, several studies have reported that the additive action is more important than the non-additive gene action in modulating the inheritance of secondary traits in tropical maize, especially under non-stress environments (Akinyosoye et al., 2025; Kavai et al., 2025; Bonkoungou et al., 2024; Luz et al., 2024; Adewale et al., 2023; Owusu et al., 2023; Olayiwola et al., 2021; Oyekale et al., 2020). Consequently, tropical maize can be improved for this large set of traits through breeding schemes that capitalize on additive gene action, such as intra-recurrent selection schemes, and the selection of the best tropical maize lines may be done based on their per se performance during the inbred line development. Additionally, promising hybrids for those traits may be selected based solely on prediction of the GCA effects of the parental lines using a single representative tester from each heterotic group (Abu et al., 2021; Ertiro et al., 2017; Badu-Apraku et al., 2016; Fasahat et al., 2016; Hallauer et al., 2010). Regarding genetic gains, the moderate ${\hat{h}}^{2}$ values presented for almost all these traits suggest the presence of reasonable genetic variability in our maize breeding germplasm, and, consequently, their genetic improvement will be successful, resulting in moderate-to-high genetic gains (Cobb et al., 2019; De La Fuente et al., 2013; Hallauer et al., 2010). Moreover, all these traits might be improved simultaneously through a multi-trait selection strategy, since no strong undesirable association was observed between them based on values of GCA effects of parental lines. Finally, as TKW and grain morphology traits were positively correlated with GY in the hybrids and were additively inherited with moderate ${\hat{h}}^{2}$ values (> 0.55), the selection of new lines for larger weight and/or size kernel using a fast high-throughput phenotyping will facilitate the development of high-yielding tropical maize hybrids (Li et al., 2025; Liang et al., 2021).

The inheritance of GY in tropical maize has been attributed to additive gene action (Akinyosoye et al., 2025; Owusu et al., 2023; Abu et al., 2021; Yu et al., 2020; Annor et al., 2019; Ertiro et al., 2017; Njeri et al., 2017) and/or non-additive gene action (Akinyosoye et al., 2025; Li et al., 2021; Annor et al., 2019; Adewale et al., 2018; Badu-Apraku et al., 2016; Makumbi et al., 2011), and the preponderance of a gene action over another depends mainly on the genetic material and tested environments. According to Sprague and Tatum (1942), in previously selected inbred lines, non-additive gene action is relatively more important than additive action in determining GY. In agreement with them, GY was largely modulated by non-additive gene action (dominance and epistatic effects) in our set of selected inbred lines. This is evidenced by the ${\hat{\sigma }}_{\text{D}}^{2}$ value much greater than ${\hat{\sigma }}_{\text{A}}^{2}$ associated with both low value of ${\hat{\sigma }}_{\text{hyb/GCA}}^{2}$ (0.26) and a strong and positive correlation between yield performance of hybrid and SCA effect ( ${\hat{r}}^{2}=0.84$) across environments. Also, the very low ${\hat{h}}^{2}$ value (0.16) for GY indicated that only a small proportion of the phenotypic variance was explained by additive genetic effects for GY in our germplasm; but non-additive gene action controlling GY may be capitalized through hybridization and reciprocal recurrent selection based on full families and testcrosses, and the selection of superior hybrids must be done based on their hybrid performance across multiple environments (Fasahat et al., 2016; Viana et al., 2013; Hallauer et al., 2010; Comstock et al., 1949). In relation to selection strategy, as GD among inbred lines was strongly positively correlated with SCA effects for GY ( ${\hat{r}}^{2}=0.64$) and yield performance of hybrid ( ${\hat{r}}^{2}=0.70$), the selection of the best parental combinations for hybrid development in tropical maize can be effectively assisted by molecular markers, avoiding crosses between genetically related inbred lines (Adewale et al., 2023; Silva et al., 2020; George et al., 2011; Bertan et al., 2007). Similarly to GY, marker-assisted selection of parental lines may also be very useful for developing early-maturing hybrids of tropical maize, since the GD among lines was negatively correlated with hybrid flowering time ( ${\hat{r}}_{\text{hybrids\_GD}}=-0.61$ and -0.66 for DTP and DTS, respectively). Therefore, early and high-yield tropical maize hybrids may be developed by crossing inbred lines with favorable GCA effects for cycle, plant stature, kernel size and weight, ED and NKR that are genetically divergent based on molecular markers information. Finally, the breeding for GY in tropical maize based on hybrids performance is also to expected to increase both NKE and EL, since these traits are also modulated by non-additive gene action and were moderately positively correlated with GY (>0.40).

Regarding heterotic grouping, the 15 inbred lines used in our study were allocated into four groups based on both the GD among lines and SCA effects for GY, and a high concordance between two approaches was observed in the placement of lines into the same group. This result indicates that both methods were very effective for grouping tropical maize lines and that the identified groups were genetically distinct. In agreement with our results, many studies have reported close concordance between heterotic groups of tropical maize inbred lines identified by SCA and SNP-based GD methods (Akinwale et al., 2014; Badu-Apraku et al., 2013, Badu-Apraku et al., 2016). They also observed that SNP-based GD method had the highest breeding efficiency for allocating inbred lines into heterotic groups aimed at developing high-yielding and stable hybrids, regardless tested environments. Conversely, some studies have reported lack of concordance between the grouping of maize inbred lines based on SCA and SNP-based on GD methods (Bonkoungou et al., 2024; Adewale et al., 2023). The differences observed between previous findings and our results may be attributed to the genetic material and the nature of gene action underlying GY inheritance. In those earlier studies, parental inbred lines derived poorly improved populations were used, with GY predominantly governed by additive gene action. In contrast, we evaluated a set of selected inbred lines derived from breeding populations developed from commercial hybrids, and GY was mainly controlled by non-additive action, resulting in a strong correlation between SCA effects and GD among lines and, consequently, a high concordance between grouping approaches. Therefore, our findings suggest that molecular markers should be used for allocating inbred lines into putative heterotic groups in tropical maize germplasm, especially when handling with large number of improved lines, reducing the number of crosses to be made and hybrids to be evaluated in the field (Obeng-Bio et al., 2020; Silva et al., 2020). Then, heterotic information based on molecular markers may be integrated with combining ability tests to facilitate the establishment and development of well-defined heterotic groups in tropical maize (Arora et al., 2024; Gonhi et al., 2024; Adewale et al., 2023; Obeng-Bio et al., 2020; Leng et al., 2019). According to Suwarno et al. (2014), groups of lines formed by maximizing GD may be a practical starting point to develop useful heterotic groups in a maize breeding program.

The GCA effect is primarily associated with additive genetic effects and reflects the number of favorable alleles that a parental inbred line contributes to a hybrid (Fasahat et al., 2016; Viana et al., 2013; Sprague and Tatum, 1942). Maize inbred lines with desirable GCA effects can therefore be used for the development of synthetic varieties, breeding populations and hybrids, as well as for recycling inbred lines and using as testers in breeding programs (Adewale et al., 2023; Ertiro et al., 2017; Hallauer et al., 2010). According to Hallauer et al. (2010), the use of F2 genetically narrow-based populations made from elite x elite lines from the same heterotic group is the most common procedure for maize line development. Thus, we propose to use the best inbred lines identified in our study to develop bi-parental breeding populations and, consequently, newly improved inbred lines, using reciprocal testers (i.e., testers from opposite heterotic groups) and, consequently, unbalanced testcrosses. The lines VML083, VML105 and VML157, allocated into same heterotic group by both SCA and GD methods, should be used to develop three bi-parental breeding populations. The VML083 has favorable alleles for earliness and reducing plant and ear height while increasing NKR, NKE and GY; the VML105 can also contribute to reduce plant and ear height due to its highly negative values for GCA effects for PH and EH. Conversely, although VML157 has unfavorable alleles for earliness and to reduce plant stature, it can contribute positively to kernel number, size and weight, and GY in the newly developed inbred lines. Then, new inbred lines derived from these three populations may be crossed with testers from opposite groups, such as lines VML016 and VML131. These testers have desirable alleles for most tested traits and are genetically divergent from VML083, VML105 and VML157. In addition to them, lines pairs VML062 and VML131 (Group III), and VML016 and VML081 (Group I) may be recombined to develop two bi-parental populations within each group; after that, their new lines could be crossed with VML157 and VML105, respectively. The VML062 carries favorable alleles for earliness and to reduce plant stature, while also increasing kernel row number and kernel number, and GY; whereas VML081 has favorable alleles to reduce cycle and increase GY. The VML134 was not included in the next breeding cycle because it does not have favorable alleles to improve cycle, plant stature and GY in tropical maize. The development of earlier-maturing and shorter-stature maize varieties is crucial for improving tolerance to low nitrogen stress, drought, stalk lodging and high plant population, mainly across tropical environments (Abu et al., 2021; Trachsel et al., 2016). Finally, all those best lines might be used to develop breeding population though crosses with other selected inbred lines from our breeding program, with the most promising crosses and testers of new lines identified by molecular markers.

In relation to hybrid performance, the experimental hybrid VML131/VML134 out-yielded the best commercial check and was the highest-yielding hybrid, with a favorable cycle and desirable plant architecture traits. Additionally, other 11 experimental hybrids out-yielded the second highest-yielding check and exhibited desirable adjusted means for almost all tested traits, particularly flowering time and plant and ear height, comparable with commercial checks used in our study. Therefore, those 12 outstanding experimental hybrids of maize should be further evaluated in on-farm across a wider range of tropical environments and management conditions in Brazil, including the winter season, low nitrogen and drought stress, and varying plant populations. Subsequently, the two best-performing hybrids may be considered for release, production and commercialization in Brazil. It is important to highlight that the highest-yielding experimental hybrids were derived from highly divergent and complementary parental lines; also, most of these hybrids were derived from parental inbred lines carrying favorable alleles for increasing GY (i.e. positive GCA effects), and the VML131 was the parental of most of the highest-yielding experimental hybrids. Thus, our results suggest that the UFV maize breeding program has been successfully in developing elite inbred lines carrying favorable alleles for key agronomic traits, as well as hybrids with agronomic performance comparable to commercial hybrids planted by Brazilian farmers, such as the checks BM709PRO2 and DKB390PRO3 (Uberti et al., 2023; Faria et al., 2022; Zuffo et al., 2021).

## Data Availability

The original contributions presented in the study are included in the article/**Supplementary Material**. Further inquiries can be directed to the corresponding author.
